# The value of AI in the Diagnosis, Treatment, and Prognosis of Malignant Lung Cancer

**DOI:** 10.3389/fradi.2022.810731

**Published:** 2022-05-06

**Authors:** Yue Wang, Haihua Cai, Yongzhu Pu, Jindan Li, Fake Yang, Conghui Yang, Long Chen, Zhanli Hu

**Affiliations:** ^1^Department of PET/CT Center, Cancer Center of Yunnan Province, Yunnan Cancer Hospital, The Third Affiliated Hospital of Kunming Medical University, Kunming, China; ^2^Lauterbur Research Center for Biomedical Imaging, Shenzhen Institutes of Advanced Technology, Chinese Academy of Sciences, Shenzhen, China

**Keywords:** lung cancer, AI, CAD, deep learning, radiomics

## Abstract

Malignant tumors is a serious public health threat. Among them, lung cancer, which has the highest fatality rate globally, has significantly endangered human health. With the development of artificial intelligence (AI) and its integration with medicine, AI research in malignant lung tumors has become critical. This article reviews the value of CAD, computer neural network deep learning, radiomics, molecular biomarkers, and digital pathology for the diagnosis, treatment, and prognosis of malignant lung tumors.

## Introduction

The GLOBOCAN2020 cancer report released by the International Agency for Research on Cancer (IARC) of the World Health Organization shows that lung cancer has become the leading cause of death from cancer in men, and the death rate among women is second only to breast cancer (1), illustrating its serious effect on human health. The rise of AI has changed traditional tumor diagnosis, treatment and prognosis strategies. AI is the process of using computers to simulate human thinking and behavior. With the introduction of machine learning algorithms and deep learning algorithms along with the rise of big data, AI is playing an increasingly important role in the medical field. The application of AI in medicine not only reduces the workload of doctors and makes the allocation of medical resources more effective but also improves the accuracy of disease diagnosis and the prognosis of patients. This article reviews the application of AI in lung cancer.

## Basic Concepts Of AI

In 1959, the scholar Ledley and others put forward the mathematical model of computer-aided diagnosis for the first time, and diagnosed a group of lung cancer cases, which pioneered the computer-aided diagnosis. In 1966, the concept of “Computer Aided Diagnosis” (CAD) was first put forward by Ledley. Before the concept of machine learning and deep learning was put forward, CAD mainly used computer technology combined with mathematical models to build models (2). Some scholars use Gaussian scale space and multi-scale Gaussian filterbank to establish a model to detect pulmonary nodules on chest radiographs, and it is concluded that the model can detect 67% of nodules, which is close to the result of manual detection of 70% (3).This result shows that CAD has a great development prospect in the field of lung cancer. Deep learning is a machine learning technology based on computer neural networks, and computer neural networks are the study of human brain morphology, neural network structure, and letter processing, using computers to build intelligent computer models similar to human brain processing information to enhance human cognitive ability. With the advent of big data, neural network learning has evolved from traditional shallow neural networks to deep neural network learning. The concept of deep learning was proposed in the paper “Reducing the dimensionality of data with neural networks” by Hinton and Salakhutdinov (4). With the development of artificial intelligence, machine learning, a branch of artificial intelligence, and deep learning, a branch of machine learning, have played an important role. The combination of machine learning and deep learning with computer-aided detection/computer-aided diagnosis (CADe/CADx) makes it not only specific to the detection of nodules but also plays an essential role in the differentiation of benign and malignant lung nodules and the classification and staging of lung cancer. At the same time, the structured report (SR) of radiology department also plays a great guiding role in the treatment decision of lung cancer, and can strengthen the communication between imaging doctors and clinicians, while quality, Data quantification and accessibility are three important factors that transform from the current format of free-text reporting (FTR) to SR (5, 6). These three factors are closely related to AI. Some scholars have tried to use machine learning or neural network model to build a model to extract complete structure from FTR, and verified its feasibility (7, 8).

## Image Processing

### Medical Image Segmentation

Medical image segmentation is an important foundation of clinical research. There are two kinds of medical image segmentation: segmentation based on local spatial features, such as information such as gray level and texture, and segmentation based on edge information (9). Some scholars segment 20 Brain, 50 Breast, 50 Lung cancer patients and 20 Spleen scans based on adaptive geographic distance (AGD) and interactive machine learning (IML) integrated by SVMs, and get better results than manual annotation (10). Besides machine learning, the research on medical image segmentation by deep learning is also very popular. Based on the Maximum Intensity Projection (MIP), some researches have improved the U-net architecture, and put forward a network named deep residual separable neural network (DRS-CNN) to segment lung tumors. After comparing it with U-net, it is concluded that DRS-CNN has higher efficiency and fewer parameters than FCN FCN, SegNet (11). These deep learning networks all face the same problem: they need a lot of manually labeled data for training. The Cycle-consistent Generative Adversarial Network (GAN) network, which consists of two networks, Generator and Discriminator, belongs to the unsupervised learning mode. The results show that the improved CycleGAN does not need manual labeling, and can overcome the noise and reach the level of manual labeling (12). In addition, LGAN based on GAN is also proved to have better performance than U-net (13). Based on deep learning, some scholars have established a model called U-Net-Generative Adversarial Network (U-Net-GAN) by using Gan strategy, using U-Net as the Generator and FCNS as discriminators, and the experimental results show that the segmentation results in chest CT are reliable. All these studies show that GAN-based segmentation model has great potential to build a higher performance segmentation model (14).

### Image Reconstruction and Fusion

PET is mainly a radioactive tracer that decays and emits positrons, and the positrons generate annihilation radiation to generate photons. Using detectors to detect photon shapes successfully makes metabolic energy imaging, so as to diagnose diseases. Imaging to meet diagnostic requirements requires a full dose of radioactive tracer, which increases the risk of radiation exposure (15), while too low a dose will increase noise and reduce image quality. Therefore, some scholars began to consider whether low-dose PET images can be used to reconstruct images that meet the diagnostic requirements. Some scholars have used Cycliw Gans to improve the image quality on PET images of low-dose lung cancer, and compared it with traditional noise reduction methods and RED-CNN and 3D-cgan, and concluded that Cycliw Gans can effectively keep the edge and SUV value (16). Other scholars have used sparse view data acquisition to realize low-dose and high-quality imaging (17). In addition, the resolution of PET is far lower than that of CT, so it needs to be fused with the anatomical structure of CT, which is beneficial to diagnosis. Some scholars try to use CNN to improve PET/CT image fusion, and the result of foreground detection accuracy is 99.29%, which shows that CNN can significantly improve image fusion (18). Some scholars put forward the recurrent fusion network (rfn), and compared it with early fusion, late fusion and high fusion, and three tumor segmentation methods, namely resnet, densenet and 3d-unet. The results show that rfn can provide more accurate segmentation (19).

## Application of AI to Diagnosis

### CAD, CNN and GAN Based Diagnosis

**Figure 1:**
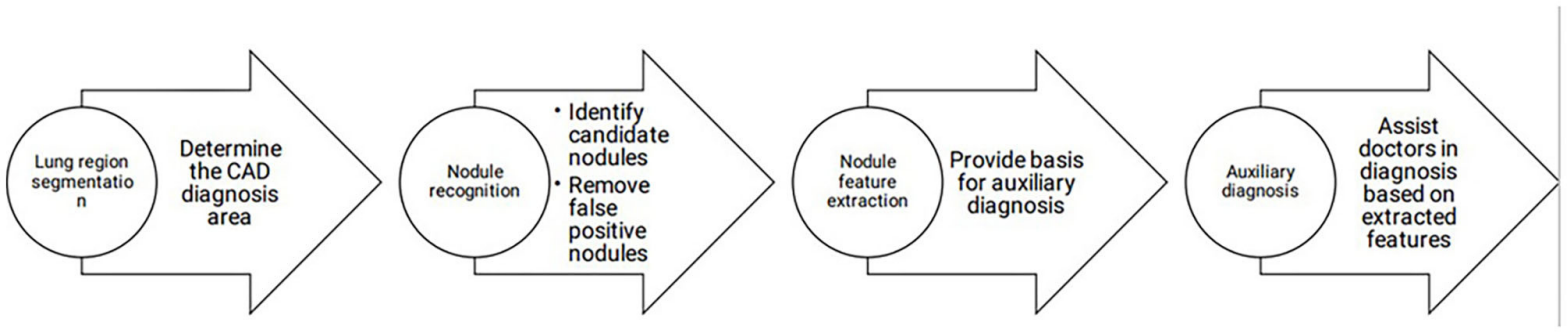
CAD workflow.

The early detection of lung cancer and early treatment are essential and related to prognostic outcomes. Facing the considerable workload associated with pulmonary nodule screening, the combination of CADe/CADx with manual diagnosis dramatically improves the efficiency of clinical work and reduces the missed diagnosis rate. At present, the detection rate of CAD for nodules is higher than that of manual diagnosis. The sensitivity is high, but the specificity of detecting nodules needs to be improved to reduce the false-positive rate (20). A schematic representation of CAD workflow is shown in Figure 1.

The combination of deep learning and lung tumor diagnosis has promoted the development of lung tumor diagnosis. AI is mainly used in lung cancer image recognition, medical image segmentation, lung nodule extraction and recognition, lung cancer pathological diagnosis, and tumor marker search in lung cancer diagnosis.

Regarding lung cancer image recognition, some scholars have used a deep convolutional neural network (CNN) to develop an automatic tumor region recognition system for lung cancer pathological images (21). Some scholars have used the Mask Regional Convolutional Neural Network (Mask R-CNN) architecture to develop a deep learning algorithm to classify cells in the lung cancer tumor microenvironment (TME) and simultaneously extract cell characteristics to predict prognosis. The results prove that the spatial organization of different cell types is predictive of patient survival and related to the gene expression of biological pathways (22), showing that AI can be used to identify tumor regions on lung cancer pathological images and to segment various cells in the lung cancer tumor microenvironment. In terms of image segmentation, medical images have a low resolution and low contrast compared with natural images. In addition, the shape of lung cancer images is irregular and can include speculation, lobulation, pleural indentation, and other shapes with unclear edges. There may also be calcified cavities and pleural effusions, increasing the difficulty of lung cancer image segmentation. The application of deep learning can improve the accuracy of image recognition and classification. In terms of lung nodule detection, compared with traditional machine learning methods, deep learning can quickly learn features of different dimensions, shorten the feature selection and calculation time, and significantly improve the efficiency of nodule detection. CNN is still being optimized. Previous studies have shown that two doctors who lack diagnostic experience could diagnose lung cancer nodules in 120 suspected lung cancer cases with nodule diameters larger than 3 mm using a CAD-aided diagnosis system based on the deep learning Faster R-CNN as the framework and not using a CAD-aided diagnosis system, respectively. The results show that after doctors use the CAD-assisted diagnosis system based on deep learning, the diagnostic sensitivity of lung cancer nodules is significantly improved, and the positive predictive value is significantly reduced. The reading time is shortened, especially for suspicious nodules with diameters of 3–6 and 6–10 mm (23), which can effectively alleviate the burden of clinical work. In terms of lung nodule detection, compared with the traditional machine learning method, deep learning can quickly learn the features of different dimensions, shorten the feature selection and calculation time, and significantly improve the efficiency of nodule detection. Among them, the convolutional neural network is a commonly used deep network. The deeper the depth of the convolutional neural network is, the richer the features of the extracted lung nodules; however, it also has shortcomings. Increasing the depth will prolong the training time of the model. In subsequent work, researchers should focus on extracting more nodule features while shortening the training time. GAN is also more and more used in the detection of lung nodules. GAN plays a very important role when only a small amount of data can't be used for deep learning model training. Studies have shown that a large number of images can be generated by GAN, and then the model can be built and trained by deep learning. The results show that this method can improve the classification accuracy by about 20% (24). This kind of transfer learning can generate a large amount of data, which solves the problems of manual annotation in deep learning and insufficient data samples (25). During the COVID-19 epidemic, many researches based on GAN achieved good results, which proved the above conclusions (26–28).

### Differentiation of Benign and Malignant Pulmonary Disease

**Figure 2:**
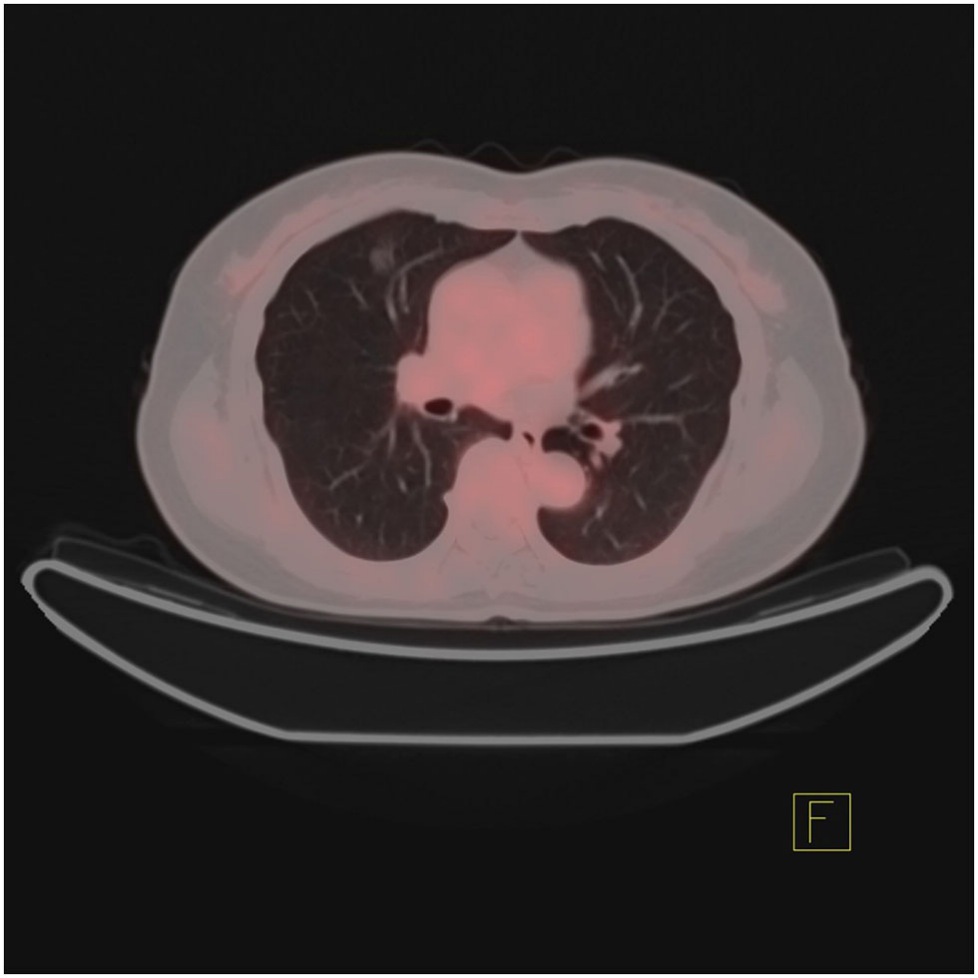
Non-metabolic invasive adenocarcinoma on PET/CT.

**Figure 3:**
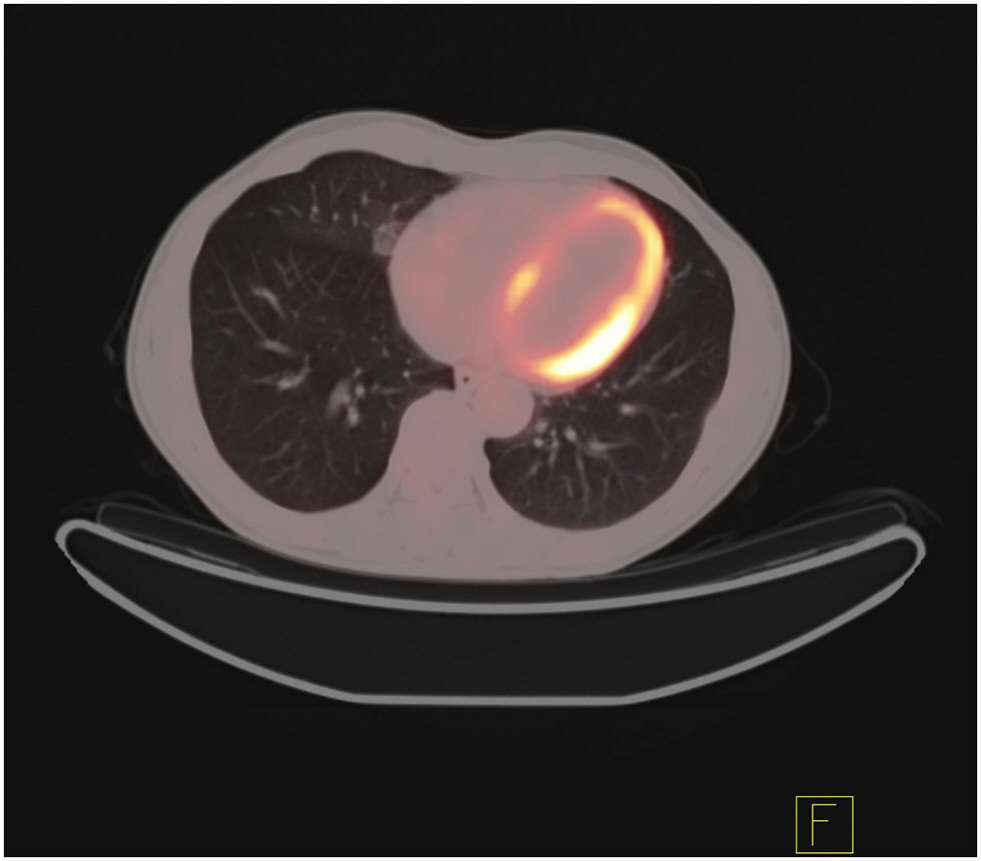
Non-metabolized granulomatous inflammation on PET/CT.

In addition, the differentiation of benign and malignant pulmonary nodules is an essential part of computer-aided diagnosis. Figures 2, 3 shows that on PET/CT, it is not easy to distinguish between benign and malignant nodules with the naked eye (Figure 2: Non-metabolic invasive adenocarcinoma on PET/CT, Figure 3: Non-metabolized granulomatous inflammation on PET/CT). For the diagnosis of benign and malignant pulmonary nodules, artificial intelligence is still dedicated to extracting certain image features, such as image size, shape, texture features, and semantic features. In machine learning, some scholars have used SVM to classify texture features, achieving an AUC of 0.9270 (29). Some scholars have used SVM to classify shape features and texture features, and the resulting AUC value was 0.9337 (30). In addition to machine learning, deep learning has also been used to identify lung nodules. Some scholars have used deep belief networks (DBNs) and CNNs to classify benign and malignant lung nodules for the first time in the classification of lung nodules. The sensitivity rates were 73.40 and 73.30%, respectively. Some scholars used a deep belief network (DBN) and CNN to classify benign and malignant lung nodules for the first time in patients with lung nodules, and the sensitivity rates were 73.40 and 73.30%, respectively (31). After that, an end-to-end framework for deep learning based on CNN was constructed, such as the multicrop convolutional neural network (MC-CNN), achieving an AUC value of 0.93 (32) and 3D CNN (33). In addition, some scholars have used CNN to establish a hierarchical semantic convolutional neural network (HSCNN) based on semantic features to detect nodules and identify nodules, and its AUC value was 0.8780 (34). Compared with machine learning, the end-to-end model of deep learning reduces the workload of data annotation. While deep learning requires much training data, to solve this problem, some scholars have proposed the transferable multimodel ensemble (TMME) algorithm (35) and Fuse-TSD algorithm (36), yielding AUC values of 0.9778 and 0.9665, respectively. According to the above research results, artificial intelligence in lung nodule identification has a good effect.

### Methods to Improve Diagnosis Accuracy

While improving the diagnostic sensitivity, reducing the false-positive rate is also an important research direction. The reason for the false-positive rate is the complex structure of the lung. In addition to lung issue and large blood vessels, it is difficult to distinguish between benign and malignant nodules because the density of inflammatory tissue is similar to the that of tumor tissue, and both show a high level of glucose metabolism. In addition, complications such as pleural adhesions and pleural effusion make it difficult to distinguish tumor tissue from normal lung tissue. The rise of radiomics has been devoted to the reduction in the false-positive rate. Some scholars have used machine learning algorithms to construct a nomogram based on the features extracted by radiomics. In the training set and validation set, the false-positive rate dropped from 30.9 to 30.4% based solely on the doctor's diagnosis to 9.1 and 5.4%, respectively (37). Some scholars used a multipath 3D CNN to build a model based on the suspicious nodules' size, shape, and background information, which significantly reduced the false-positive rate (38). Their research shows that the false-positive rate can be reduced when AI is combined with medical imaging.

### Trying to Detect Histological Types

The results of pathological diagnosis are critical to the final treatment decision and prognosis. It takes considerable time and energy for doctors to make a pathological diagnosis. Some scholars use CNN based on the EfficientNet-B3 architecture to establish a model to predict whether pathological images indicate lung cancer (39). In addition, since 80% of lung adenocarcinomas have multiple histological types, identifying histological types is very important for guiding the treatment choice. Therefore, some scholars have used deep learning convolutional nerves to classify lung adenocarcinoma types for the first (40). The above studies have achieved good results, indicating that AI can be combined with pathological diagnosis, reducing the burden on doctors and improving the accuracy of diagnosis. In terms of finding tumor markers, liquid biopsy has the advantages of noninvasiveness, real-time dynamic detection, and repeatability. However, the content of detected substances in the liquid biopsy is not high, so the detection sensitivity is not high. The use of machine learning algorithms to develop models that interpret the signals in the samples can effectively improve the detection sensitivity. The application of AI in liquid detection includes Foundation One based on cancer genomics (41).

## The Role of Artificial Intelligence in Therapy

### Targeted Therapy

The histological classification of lung cancer is mainly divided into small-cell lung cancer (SCLC) and non-small-cell lung cancer (NSCLC). Among them, non-small-cell lung cancer accounts for 85% and is further divided into squamous cell carcinoma, adenocarcinoma, squamous adenocarcinoma, and large cell lung cancer (42). For lung cancer treatment, the conventional treatment methods include surgical resection, radiotherapy, and chemotherapy. Targeted therapy also plays an increasingly important role due to its individualized treatment, excellent curative effect, and relatively few adverse reactions. Targeted therapy refers to targeting drugs that act on receptor proteins, enzymes, and genes during tumor cell proliferation to disrupt cell growth. Among the current targeted therapies, non-small-cell lung cancer targets include EGFR (epidermal growth factor receptor), EML4-ALK fusion gene (43), ROS1 fusion gene, RAS mutation, and C-MET amplification. In the process of targeted therapy, the most critical step is to determine the corresponding target. The primary traditional detection method is sequencing. However, heterogeneity within the tumor may cause inaccurate results for sampling and sequencing (44).

**Figure 4:**
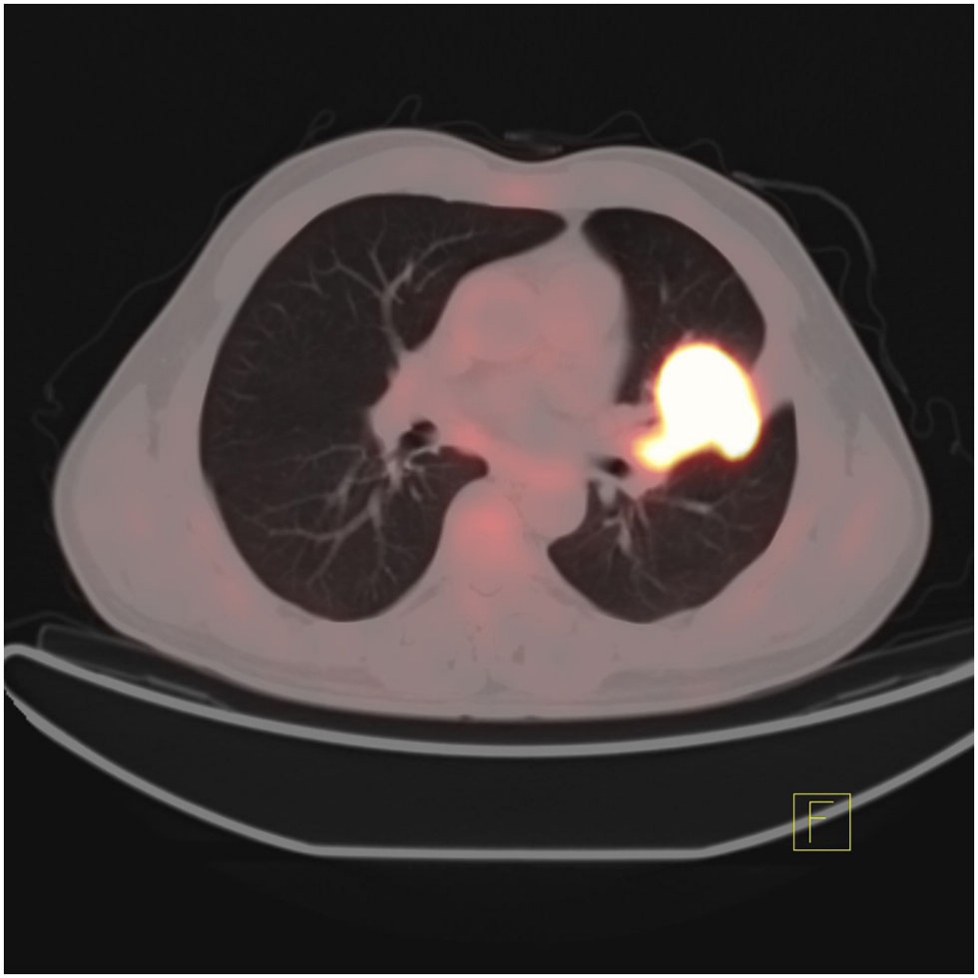
PET/CT manifestations of lung adenocarcinoma patients with EGFR mutation.

**Figure 5:**
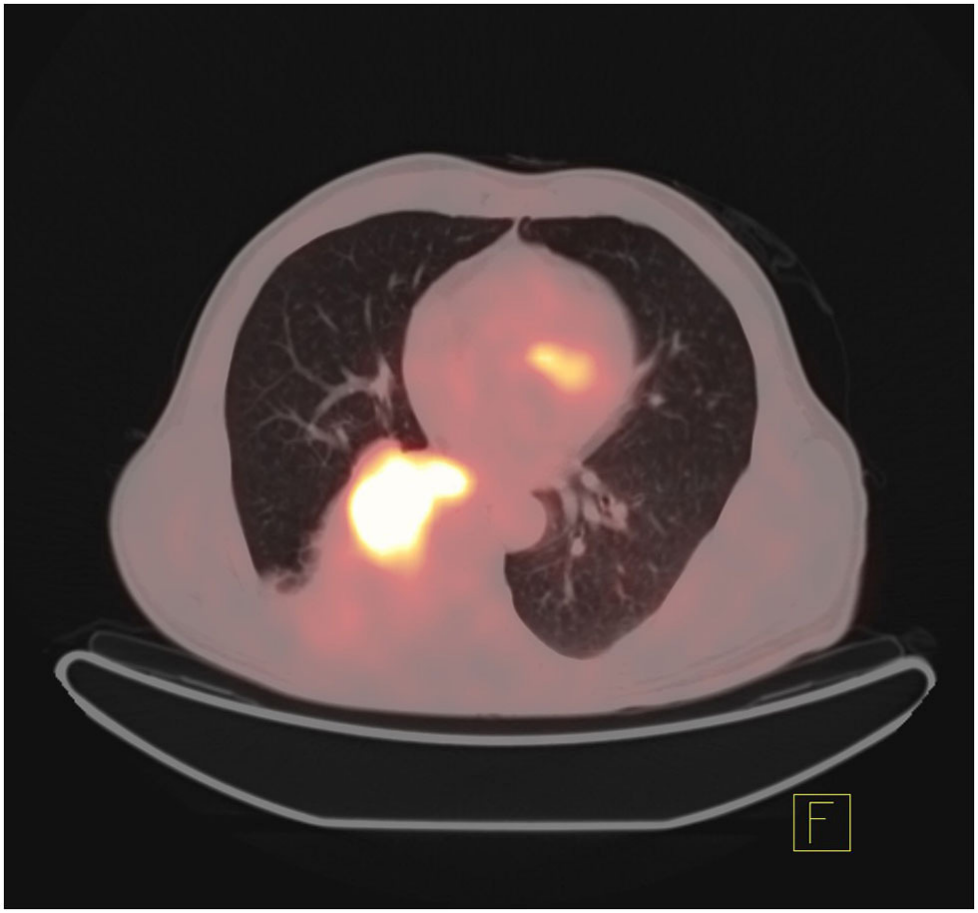
PET/CT manifestations of EGFR wild-type patients with lung adenocarcinoma.

At the same time, surgery is invasive and may cause tumor metastasis, so it is not conducive to the repeated detection of targeted genes during the later stage of disease development. In addition to the fact that biopsy cannot be performed in all clinical cases (45), some patients may lose the opportunity for targeted therapy because they cannot undergo biopsy. In recent years, to achieve noninvasive, simple, and rapid detection, some scholars have studied the combination of radiomics and deep learning in artificial intelligence with CT and PET images to predict gene mutations. Figures 4, 5 show that it is not easy to distinguish between EGFR mutant type and wild type on PET/CT (Figure 4: EGFR mutant, Figure 5: EGFR wild type). In terms of radiomics, some scholars have used radiomics to extract the features of PET/CT images and used logistic regression to establish a model to predict EGFR mutations. The AUC values of the training set and validation set were 0.79 and 0.85, respectively. At the same time, scholars also established a clinical model based on sex and smoking history, with AUC values of 0.75 and 0.69 obtained for the training set and the validation set, respectively. The AUC values of the training set and the validation set of the combination of radiomics and clinical models were 0.86 and 0.87, respectively (45). In addition, some scholars have used a multivariate random forest algorithm and logistic regression model to screen out the image features most relevant to EGFR mutations on segmented PET/CT images and then employed the supervised XGBoost machine learning algorithm to establish a prediction model of EGFR mutation subtypes. The results were as follows: AUC value for exon 19 deletion was 0.77, the AUC value for exon 21 L858R point mutation was 0.92, and the overall model AUG for EGFR mutation was 0.87 (46). Scholars have also compared the performance of the gradient tree boosting algorithm and random forest algorithm (RF) to predict lung cancer subtypes and EGFR mutations. Combining the above two algorithms, the gradient tree boosting algorithm was obtained by comparing the AUC values. The conclusion was that the gradient tree boosting predictive algorithm ability is better than the random forest algorithm (47). In addition, machine learning algorithms such as SVM are used to predict target mutations (48). Because the use of machine learning to build a model requires precise manual segmentation steps, to simplify the delineation step, some scholars have proposed applying the end-to-end learning of deep learning to the prediction of EGFR mutation status. Deep learning models such as 2D small residual convolutional network (SResCNN) models and the use of 3D CNNs to develop 3D DenseNets have been established with the development of deep learning. However, the current research generally has certain problems, such as insufficient experimental samples (49, 50).

### Immune Therapy

**Figure 6:**
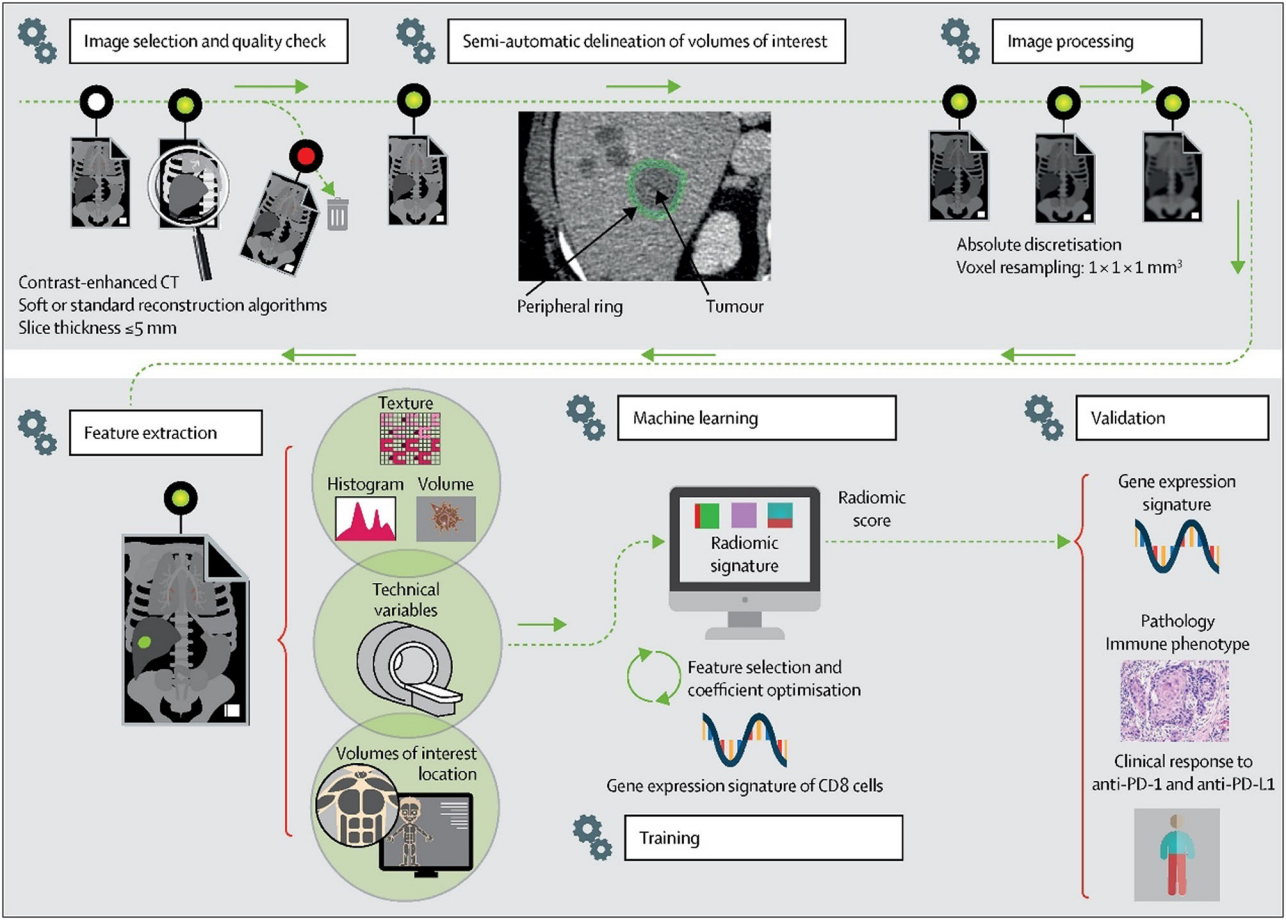
Radiomics workflow.

In addition, an immune checkpoint inhibitor (ICI) is also an effective method for treating lung cancer. Immunotherapy uses ICIs to restore the immune response toward tumor cells in order to slow the growth of tumors. At present, ICIs mainly act on the PD-1/PDL-1 pathway in lung cancer. Although immunotherapy has made significant progress in the treatment of lung cancer, ICIs are not practical for all patients and are effective in <30% of lung cancer patients (51). Therefore, it is similar to targeted therapy in that it is also necessary to detect the amount of PD-L1 protein in tumor tissue through biopsy and immunohistochemistry before immunotherapy. Unfortunately, biopsy in this case has the same problems as the targeted therapy biopsy above. Some scholars have used gene sequencing to detect the abundance of CD8 cells combined with enhanced CT generation to evaluate the imaging characteristics of CD8 cell tumor invasion and then used linear ElasticNet regression in machine learning to develop CD8 cell expression signature markers based on radiomics. In a study aiming to predict the tumor immunophenotype in order to determine whether ICI is effective and to predict the treatment response after receiving ICI treatment, the AUC value was found to be 0.74 for the expression classification of CD8 (high abundance vs. low abundance) and gene expression in CD8 cells. The characteristic AUC was 0.67 (52). Some scholars have combined CT imaging radiomics and clinical information to predict the expression of PD-L1, and the AUC in the prediction verification set was 0.848 (53). In addition, some scholars have used clinical data and radiomics of PET/CT images in combination with SResCNN of deep learning to establish a PD-L1 prediction model. They also used this model to predict the prognosis of patients for immunotherapy and achieved good results, with an AUC of 0.82 (54). In addition to radiomics, some scholars have also tested the RNA expression level of patients with relapsed NSCLC who received PD1/PD-L1 treatment and used the obtained genome to use machine learning to perform feature selection and establish a prediction model to accurately predict whether the patient is suitable for anti-PDL-1 treatment (55). Some scholars believe that clinical characteristics such as PD-1 cannot independently and accurately predict whether ICIs are beneficial to patients. Therefore, after comparing various machine learning algorithms, such as GBM and XGBoost, they chose the LighGBM algorithm to build a comprehensive prediction model for multiple clinical features, including patient characteristics, laboratory results, tumor size, genetic mutation status, metastatic location, treatment route, and PD-1 inhibitor type and response, yielding an AUC value of 0.788, which was better than the prediction model established by a single clinical feature (56). The above experimental results show that the combination of artificial intelligence and immunotherapy can help to establish a better predictive model to make clinical treatment decisions that are most beneficial to patients. The schematic diagram of the radiomics workflow is shown in Figure 6 (52).

### Radiotheraphy

In the treatment of early lung cancer, especially in patients who cannot be operated on, stereotactic ablative radiotherapy (SABR) plays an important role, which the 3-year survival rate of patients with inoperable non-small cell lung cancer who received SABR was 55.8% (57). With the in-depth study of artificial intelligence in image segmentation, some scholars have tried to develop an automatic lung segmentation model in ablation radiotherapy through deep learning. Through the author's research, they found that although the current segmentation results are not comparable to the manual segmentation results, their research Proved that this is feasible (58). At the same time, some scholars used Deep Profiler in the deep learning neural network to predict the image fingerprint of CT images based on the purpose of precision treatment, and found that the failure rate of radiotherapy for patients with low scores was significantly lower than that for patients with high scores (59). While SABR brings benefits to patients, it also has corresponding side effects, such as Radiation-induced lung injury (RILI) (60), Chest wall pain and rib fractures, etc. (61). In order to predict the occurrence of side effects, some scholars have conducted research. Especially for the prediction of RILI, scholars have used AI to make predictions from various angles: For the first time, scholars extracted radiomic features from the gross tumor volume (GTV) in CT images of patients receiving treatment from two different medical institutions, and used the extracted radiomic features and clinical/dose to establish regularized models to predict local lung fibrosis (LF) about time and frequency of occurrence, but the model is not applicable to the prediction of disease-free survival (DFS) and overall survival (OS) (62). Based on the data of 3 medical institutions, some scholars have used LightGBM to develop three different models from the perspectives of dose-volume indices (DVIs), radiomics and a mixture of the two to predict radiation pneumonia of grade 2 and above, and the ROC of the three models. ROC-AUC and PR-AUC values are 0.660 ± 0.054 and 0.272 ± 0.052, 0.837 ± 0.054 and 0.510 ± 0.115 and 0.846 ± 0.049 and 0.531 ± 0.116, respectively (63). The above results indicate that there is still a lot of room for the development of AI in the related fields of SABR treatment of lung cancer.

## The Role of Artificial Intelligence in Predicting the Prognosis of Lung Tumors

An accurate prognosis can guide the selection of the most beneficial treatment for lung cancer patients. The treatment and prognosis of patients are affected by TNM staging. With the development of artificial intelligence, lung cancer has made great progress in diagnosis and treatment, but at the same time, people can also provide more accurate prognoses through artificial intelligence to make decisions for patients based on the prediction results. The most beneficial treatment decisions can help to prevent unnecessary treatment. In terms of complications, some scholars have used artificial neural networks (ANNs) to establish a prediction model for the occurrence of fungal lung infections in lung cancer patients based on seven variables: age, antibiotic use, low serum albumin concentrations, radiation therapy, surgery, low hemoglobin hyperlipidemia, and length of stay in hospital. The AUC value was 0.829 ± 0.019 (64). Some scholars proposed medical MLP (MediMLP) based on the multilayer perception model (MLP). This model uses the Grad-CAMA algorithm for feature selection to determine whether complications will occur after lung cancer surgery as well as the types of complications (lung, other organs, whole body); the results indicated that the postoperative indwelling drainage tube time is the key to whether complications occur (65). Some scholars use wearable activity trackers to collect patient-generated health data (PGHD) and patient-reported outcomes (PROs) after discharge from the hospital and use the L2 regularized logistic regression model to predict whether complications will occur during the early postoperative period. In that study, the prediction of complications after discharge was most relevant to the length of hospital stay and complications before discharge. This regular remote detection model is the first model to predict complications after discharge. It was found to improve the survival rate and more effectively use medical resources (66). In addition, in terms of lung cancer patient survival and risk stratification, some scholars have used clinical data and radiomics of PET/CT images combined with SResCNN in deep learning to establish a PD-L1 immunotherapy prediction model. The deep learning score (DLS) predicted persistent clinical benefit (DCB), overall survival (OS), and progression-free survival (PFS). The conclusion was that DLS combined with clinical features can accurately predict DCB, OS, and PFS (54). Some scholars established an immune prognosis model composed of two immune genes (ANLN and F2) by using LASSO and Cox regression analysis along with the software CIBERSORT to analyze immune infiltration based on the differential expression of mRNA in tumor vs. normal tissues and predicted patients with a high risk of a poor prognosis. At the same time, the model and pathological analysis were combined to establish a nomogram for prognosis. The experimental results show that the comprehensively established nomogram has a higher AUC for prediction (67). The DeepSurv model, which aimed to evaluate patient prognosis and treatment recommendations and was composed of clinical information (sex, age, marital status), tumor characteristics (location, size, histological type and grade, and TNM staging), and treatment details from the SEER database and based on a deep feed-forward neural network and the Cox proportional hazard model, was found to have a better predictive ability than TNM. At the same time, it also developed the user input view, the survival prediction view, and the treatment recommendation view to visualize the DeepSurv model (68). Some scholars use the EfficientNet model to segment tumor cells and tumor-infiltrating cells in tumor microarrays (TMAs). At the same time, ResNet was used on the immunohistochemical images to extract the characteristics of OS and RFS (recurrence-free survival), and a reliable prediction model was established (69). Some scholars have used WGCNA to analyze the gene modules related to hypoxia based on the imbalance between the supply and demand of the hypoxia response tumor environment and then use the LASSO Cox algorithm or logistic regression model and RF to screen out the most relevant hypoxia prognostic genes, establish formulas, and calculate the hypoxia-related prognostic risk score (HPRS) of the sample. They concluded that the higher the HPRS is, the worse the OS of the patient. At the same time, a multivariate Cox regression analysis with age, stage, and sex demonstrated the excellent predictive ability of HPRS, and the use of HPRS and clinicopathological characteristics to establish a predictive nomogram achieved high accuracy (70). To date, a prognostic model of lung cancer has been established based on radiomics, genomics, clinical features, and pathological results. The established model has a good predictive ability from the experimental data, which is significant for guiding the choice of lung cancer treatment. Table 1 shows the development of AI in the field of lung cancer.

**Table 1:** The development of AI in the field of lung cancer.

**Author**	**Model**	**Function**	**AUC**	**Sensitivity**
Han et al. (29)	SVM	To identify benign and malignant nodules based on texture features	0.9270	–
Dhara et al. (30)	SVM	To identify benign and malignant nodules based on texture and shape features	0.9337	–
Yu-Jen et al. (31)	DBN	To identify benign and malignant nodules for the first time	–	73.40%
	CNN		–	73.30%
Xie et al. (36)	TIME algorithm	To distinguish benign and evil nodules	0.9778	–
	Fuse-TSD algorithm		0.9665	–
Fei Kang et al. (37)	Machine learning combined with radiomics	To construct a nomogram to reduce the false-positive rate	0.98	–
Zhang et al. (45)	Logistic regression	To establish an EGFR mutation prediction model based on PET/CT radiomics and clinical data	0.87	–
Qiu et al. (46)	RF. logistic regression model xgboost machine learning algorithm	To establish a prediction model of EGFR mutation subtypes and EGFR status	EGFR exon 19 deletions 0.77	–
			EGFR exon 21 L858R missense 0.92	–
			EGFR mutation positivity 0.87	–
Koyasu et al. (47)	XGB algorithm combined with Bayesian algorithm	To predict EGFR mutation status based on radiomics	0.659	–
	RF algorithm combined with Bayesian algorithm	To predict of EGFR mutation status based on radiomics	0.577	–
Sun et al. (53)	Combination of CT radiomics and clinical information	To predict PD-L1 expression	0.848	–
Wiesweg et al. (55)	Detect the RNA expression level of tumor immune context, use machine learning	To establish a predictive model to predict whether it is suitable for anti-PDL-1 therapy	–	–
Ahn et al. (56)	LighGBM algorithm	To establish a predictive anti-PD-1 response model based on multiple clinical features	0.788	–
Chen et al. (64)	ANN	To predict the occurrence of lung fungal infections in lung cancer based on clinical information	0.829 ± 0.019	–
He et al. (65)	MediMLP model	To predict postoperative complications and types of lung cancer	0.88	–
Rossi et al. (66)	L2 regularized logistic regression	To predict whether complications will occur in the early postoperative period for the first time	0.74	–
Luo et al. (67)	LASSO and COX regression analysis and CIBERSORT	To establish an immune prognostic model and nomogram according to the differential expression of mRNA between tumor and normal tissues to identify high-risk patients	0.7061	–
She et al. (68)	Deep feed-forward neural network and the Cox proportional hazard model	To evaluate patient prognosis and treatment recommendations, the DeepSurv model was established	–	–
Guo et al. (69)	The EfficientNet model and ResNet	To build a predictive model on immunohistochemical images	0.913	–
Shi et al. (70)	LASSO Cox algorithm	To calculate the HPRS and HIRS of the sample and establish a nomogram	0.809	–

## Discussion

For the diagnosis of pulmonary nodules, the application of AI has improved the detection rate, but we should also focus on improving the specificity of nodules to reduce the false-positive rate. For images of lung cancer tumors, confidential information is not visible to the naked eye, but with the help of AI, radiomics, and genomics, we can extract this information and combine it with clinical information for patient diagnosis, treatment, and prognosis. The current research still has the following problems. At present, most AI models are tested in a single race. Data from multiple sources should be tested to determine whether it can be applied to other races (71). In addition, the prediction or classification model established based on deep learning has black-box attributes because the neural network is not interpretable. Thus, we should further study how the neural network model calculates and make judgments. At the same time, we should explore the specific relationship between deep learning and radiomics to establish a better model. According to previous studies (45, 56), the combination of clinical features, semantic features, radiomics, and deep learning has better prediction or classification capabilities than models established by a single factor. Therefore, we should continue to explore how the above factors can be combined to establish the best model in the future. The development of AI and the rise of big data are inseparable; standardizing effective and sufficient data is essential for deep learning model training. Therefore, in the future, a standardized and shared database should be established for research so that artificial intelligence can be better used in lung cancer diagnosis, treatment, and prognosis. Finally, it is worth mentioning that although the application of artificial intelligence in medical care has a great development space, it also faces many other problems, such as ethical, psychological and legal issues. With the development and application of AI, especially when AI is used to decide the end of life in the future, perhaps we need new principles and regulations to manage medical artificial intelligence, so as to make the most favorable decision for patients and achieve the goal of “doing no harm” (72, 73). In order to achieve this goal, doctors should not accept the combination of AI with medical care without criticism, nor should they resist the application of AI without reason, but should actively participate in and promote its development (74). In addition, on the premise that deep learning has black-box attributes, we should not ignore patients' fear, but also ensure patients' right of informed consent and explain the benefits and potential risks, so as to solve patients' excessive fear or overconfidence (75). When AI is applied to medical disputes, the division of responsibilities may involve doctors, artificial intelligence system manufacturers and regulatory agencies (76), so relevant laws and regulations must be actively improved to solve related disputes.

## Author Contributions

ZH, LC, and YW conceived and designed the study. YW, HC, YP, FY, JL, FY, and CY retrieved and analyzed the documents. YP collected the figures. YW wrote the manuscript. ZH and LC supervised the study, reviewed, and edited the manuscript. All authors approved the final manuscript.

## Funding

This study was supported by the Project funded by China Postdoctoral Science Foundation (No. 2019M653501), the National Natural Science Foundation of China (No. 81960496), Yunnan Fundamental Research Projects (No. 202101AT070050), the Funding Project of Oriented Postdoctoral Training in Yunnan Province, the 100 Young and Middle-aged Academic and Technical Backbone Incubation Projects of Kunming Medical University, Reserve candidates for Kunming's young and middle-aged academic and technical leaders (17th), and Yunnan health training project of high-level talents (H-2018006) and Top Young Talents in Yunnan Ten Thousand Talents Program (2020).

## Conflict of Interest

The authors declare that the research was conducted in the absence of any commercial or financial relationships that could be construed as a potential conflict of interest.

## Publisher's Note

All claims expressed in this article are solely those of the authors and do not necessarily represent those of their affiliated organizations, or those of the publisher, the editors and the reviewers. Any product that may be evaluated in this article, or claim that may be made by its manufacturer, is not guaranteed or endorsed by the publisher.
